# Cloning and Functional Analysis of Dwarf Gene *Mini Plant 1* (*MNP1*) in *Medicago truncatula*

**DOI:** 10.3390/ijms21144968

**Published:** 2020-07-14

**Authors:** Shiqi Guo, Xiaojia Zhang, Quanzi Bai, Weiyue Zhao, Yuegenwang Fang, Shaoli Zhou, Baolin Zhao, Liangliang He, Jianghua Chen

**Affiliations:** 1 CAS Key Laboratory of Tropical Plant Resources and Sustainable Use, Xishuangbanna Tropical Botanical Garden, Chinese Academy of Sciences, 88 Xuefu Road, Kunming 650223, China; guoshiqi17@mails.ucas.ac.cn (S.G.); zhangxiaojia0512@163.com (X.Z.); baiquanzi@xtbg.ac.cn (Q.B.); zhaoweiyue@xtbg.ac.cn (W.Z.); fangyuegw@163.com (Y.F.); zhoushaoli@xtbg.ac.cn (S.Z.); zhaobaolin@xtbg.ac.cn (B.Z.); 2 College of Life Science, University of Chinese Academy of Sciences, Beijing 100049, China

**Keywords:** dwarfism, gene cloning, *MNP1*, CPS, *Medicago truncatula*

## Abstract

Plant height is a vital agronomic trait that greatly determines crop yields because of the close relationship between plant height and lodging resistance. Legumes play a unique role in the worldwide agriculture; however, little attention has been given to the molecular basis of their height. Here, we characterized the first dwarf mutant *mini plant 1* (*mnp1*) of the model legume plant *Medicago truncatula*. Our study found that both cell length and the cell number of internodes were reduced in a *mnp1* mutant. Using the forward genetic screening and subsequent whole-genome resequencing approach, we cloned the *MNP1* gene and found that it encodes a putative copalyl diphosphate synthase (CPS) implicated in the first step of gibberellin (GA) biosynthesis. MNP1 was highly homologous to *Pisum sativum* LS. The subcellular localization showed that MNP1 was located in the chloroplast. Further analysis indicated that GA_3_ could significantly restore the plant height of *mnp1-1*, and expression of *MNP1* in a *cps1* mutant of *Arabidopsis* partially rescued its mini-plant phenotype, indicating the conservation function of MNP1 in GA biosynthesis. Our results provide valuable information for understanding the genetic regulation of plant height in *M. truncatula.*

## 1. Introduction

Dwarf phenotypes have been widely used to improve lodging resistance and enhance harvest index in crops. For this reason, the proper modulation of plant height has always been a priority for breeders. Although many factors regulate plant height, gibberellin (GA) plays a leading role and is also known as the “green revolution phytohormone” because of its great contribution to the cultivation of high yields and lodging resistant crop varieties. The “green revolution” gene *semi-dwarf 1* (*sd1*) encoding GA biosynthesis enzyme GA 20-oxidase (GA20ox) is always important in rice breeding from the 1960s [1]. The *reduced height 1* (*rht-B1b* and *rht-D1b*) mutants showed a semi-dwarfing phenotype due to insensitivity to GA and were also used to breed for lodging resistance and yield increase in wheat [2].

GA is involved in various processes of plant growth and development, including leaf expansion, seed germination, induction of flowering and stem elongation [3,4,5,6,7]. With the extensive characterization of dwarf mutants related to GA, numerous genes encoding GA biosynthetic enzymes have been identified [8,9]. Bioactive GA biosynthesis is divided into three stages. In the first stage, geranylgeranyl diphosphate (GGDP), the precursor of GA, is catalyzed by the copalyl diphosphate synthase (CPS) and *ent*-kaurene synthase (KS) to form *ent*-kaurene, and this process takes place in the plastid [10,11]. Then, in the second stage, *ent*-kaurene is converted to GA_12_ by *ent*-kaurene oxidase (KO) and *ent*-kaurenoic acid oxidase (KAO), both of which are cytochrome P450 enzymes [11,12,13]. In the final stage, GA_12_ is catalyzed by GA20ox and converted to GA_9_ via GA_15_ and GA_24_, and then GA_9_ is converted to GA_4_ by GA 3β-hydroxylase (GA3ox) [14,15,16,17]. GA_12_ is also converted to GA_1_ through the 13-hydroxylation pathway [9]. The biosynthesis of bioactive GA_1_ and GA_4_ occurs in the cytoplasm. Because the early-step genes of GA biosynthesis, *CPS1/GA1*, *KS* and *KO*, are single copy in *Arabidopsis*, mutations of these genes usually induce severely dwarf phenotype with greatly impaired fertility [12,18]. In contrast, the loss-of-function mutants of *GA20ox* and *GA3ox* (the late-step genes of GA biosynthesis) show a semi-dwarf phenotype due to the functional redundancy of multiple copies of genes [19,20]. In addition, the mutants altered in GA degradation and signal transduction pathway also show various degrees of dwarf phenotype and are valuable in molecular breeding [21,22,23,24,25].

Previous studies have suggested that, in addition to GAs, other plant hormones, such as brassinosteroids (BRs) [26,27] and strigolactones (SLs) [28,29], also play important roles in plant height development. The dwarf mutants related to these hormones can be divided into two types, hormone-sensitive and hormone-insensitive. The plant height of those hormone-sensitive mutants could be restored by exogenous hormones because their hormone content is reduced due to the disorder of hormone metabolic pathways [26,29,30,31,32]. The hormone-insensitive mutants are not sensitive to the hormone due to the abnormal signaling pathway [27,33,34,35,36]. So far, the molecular genetic pathways underlying the plant height regulation are well-characterized in *Arabidopsis* and rice, but only a few studies have been conducted on other species.

Legumes are the second most important economic crops after cereals and provide the major sources of plant proteins and oils for humans and animals [37]. Investigations on dwarf mutants in peas and soybeans have strongly suggested that the GA pathway plays a conserved role in determining the plant height of legumes [38,39,40]. *Medicago truncatula*, a diploid model legume plant, has been sequenced [41,42], but little attention was given to the basis of its height and the involved regulatory mechanisms of the GA pathway.

In this study, we characterized the severely dwarf mutant *mnp1* with two alleles isolated from the *Tnt1* retrotransposon-tagged mutant population of *M. truncatula*. Through forward genetic screening and the subsequent whole-genome resequencing approach, we cloned the *MNP1/Medtr7g011663* gene and found that it was well-clustered with the homologous genes encoding *Pisum sativum* LS, *Solanum lycopersicum* GIB-1, *Arabidopsis thaliana* CPS1/GA1, *Oryza sativa* OsCPS1 and *Zea mays* An1, all of which are the enzymes involved in the first step of GA biosynthesis. Because the dwarf phenotype of *mnp1* was significantly restored by exogenous application of GA_3_, and the mini-plant phenotype of the *Arabidopsis cps1* mutant was partially rescued by the expression of *MNP1*, we proposed a conserved function of MNP1 in GA biosynthesis. Given the evidence that both the *mnp1* and the pea *ls* mutants are fertile and there are multiple possible copies of *MNP1*/*LS* in *M. truncatula*, peas and soybeans, it is reasonable to hypothesize that the duplication of *CPS* genes and the subsequent functional divergence may have occurred in legumes during evolution [43]. The result has significant implications for the legume breeding programs and provides a good model to further study the regulatory mechanism of height regulation in *M. truncatula*.

## 2. Results

### 2.1. Mini Plant 1 Mutants Were Severely Dwarfed Due to Shorter and Fewer Cells

**Figure 1:**
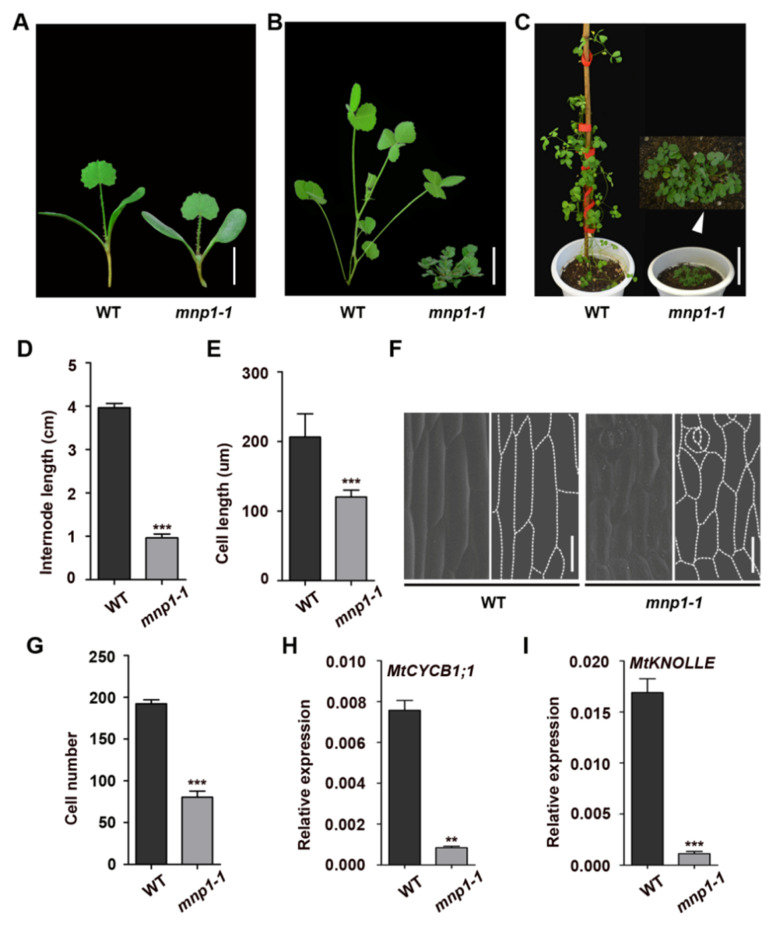
Phenotypic characterization of *mnp1-1* mutant. (**A**–**C**) Morphologies of wild type (WT) and *mnp1-1* mutant at different developmental stages. (**A**) Ten days after sowing. Scale bar = 0.75 cm. (**B**) Six weeks after sowing. Scale bar = 2 cm. (**C**) The reproductive stage of plants. Scale bar = 6.5 cm. (**D**) The length of the third internode beneath the shoot apex. Values are means ± *SD* (*n* = 20 internodes). Two-sample *t*-test, *** *p* < 0.001. (**E**) The length of epidermal cells of the third internode beneath the shoot apex. Values are means ± *SD* (*n* = 20 cells from three biological replicates). Two-sample *t*-test, *** *p* < 0.001. (**F**) Scanning electron microscope images and cell outlines of a representative third internode beneath the shoot apex. Scale bar = 50 um. (**G**) Number of epidermal cells in the third internode beneath the shoot apex. The cell number was calculated from the ratio of the average internode length (**D**) to the average cell length (**E**). Error bars represent the standard deviation of the cell number of 20 independent internodes. Two-sample *t*-test, *** *p* < 0.001. (**H**) Expression analysis of cell division marker gene *MtCYCB1;1.* Values are means ± *SD*. Two-sample *t*-test, ** *p* < 0.01. (**I**) Expression analysis of cell division marker gene *MtKNOLLE.* Values are means ± *SD*. Two-sample *t*-test, *** *p* < 0.001.

**Figure 2:**
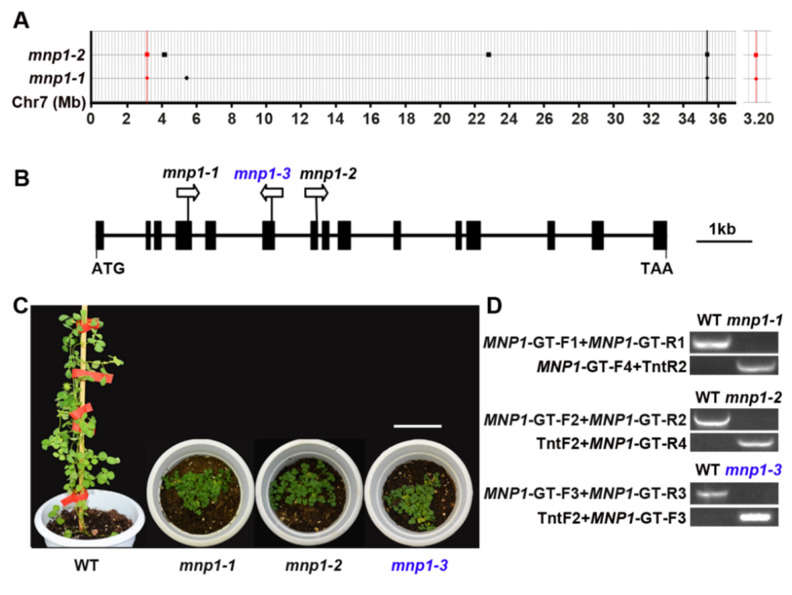
Molecular cloning of the *MNP1* gene. (**A**) Adjacent *Tnt1* insertion sites were found on chromosome 7 of *mnp1-1* and *mnp1-2*. The *x*-axis represents chromosome 7. Rhombus and squares represent *Tnt1* insertions in *mnp1-1* and *mnp1-2*, respectively. The rhombus and square on a black line show nearby *Tnt1* insertions in an intergenic region. The rhombus and square on a red line show nearby *Tnt1* insertions in *Medtr7g011663* and the right image is an enlarged view in the same region. (**B**) Schematic illustration of *MNP1* gene structure and *Tnt1* insertion sites in *mnp1* alleles. The *mnp1-3* (blue color) mutants were screened from the *Tnt1* population using a reverse genetics approach. Filled black boxes represent exons and lines between them denote introns. Arrows indicate *Tnt1* orientation. (**C**) The phenotype of *mnp1* alleles. Scale bar = 6.5 cm. (**D**) Genotyping of *mnp1* alleles. The primers (*MNP1*-GT-F/R) were designed for detecting *MNP1* genomic fragments, and the primer pair TntF2/R2 were *Tnt1*-specific primers.

Legumes are the third largest family of angiosperms, including many important crops, such as soybeans and peanuts [44]. To gain a better understanding of the molecular basis of plant height regulation in legumes, we screened the *Tnt1* retrotransposon insertion mutant collection of the model plant *M. truncatula* [45] to isolate mutants with significant changes in plant height. Two allelic mutants with similar severely dwarf phenotypes were identified and designated as *mini plant 1-1* (*mnp1-1*) and *mini plant 1-2* (*mnp1-2*), respectively, because all F_1_ progenies derived from a cross between *mnp1-1* and *mnp1-2* were dwarf plants. Compared with the wild type, the mutants are severely dwarf, with increased branches and dark green leaves (Figure 1A–C and Figure 2C).

During the growth and development from seedlings to adult plants, the height gap between the wild type and *mnp1-1* mutant was becoming bigger (Figure 1A–C). By measuring the length of the third internode beneath the shoot apex, we confirmed that the *mnp1-1* mutants have reduced internode length compared with the wild type (Figure 1D). Then, scanning electron microscopy (SEM) analysis was used to determine the reasons for the shorter internode of *mnp1-1* mutants. The epidermal cells of the *mnp1-1* internode were considerably shorter than those of the wild type (Figure 1E,F). In addition, the number of internode cells was also greatly reduced in *mnp1-1* mutants (Figure 1G), indicating that cell division was significantly suppressed. This speculation would be in agreement with the quantitative analysis of the reduced cell cycle activity of the *mnp1-1* internode. The expression of the G2/M phase cell cycle marker *MtCYCB1;1* and the cytokinesis marker *MtKNOLLE* [46] were both dramatically lower in *mnp1-1* than that of wild type (Figure 1H,I). Therefore, both decreased length and number of internode cells contributed to the shortened stem of *mnp1-1*. In addition to the decrease of stem length, the petiole of *mnp1-1* was shortened as well (Figure S1). In conclusion, these results demonstrated that *MNP1* plays an important role in the length determination of stem and petiole in *M. truncatula*.

### 2.2. Molecular Cloning of MNP1 Gene

Analysis of the F_2_ generation resulting from a cross between *mnp1-2* and wild type showed a segregation ratio of 3:1 between wild-type-like and dwarf phenotypes (36:13, χ^2^ = 0.0068 < χ^2^_0.05_ = 3.84) (Figure S2A), indicating that the *mnp1-2* phenotype was controlled by a single recessive gene. To clone the target gene corresponding for the mutant phenotype, *mnp1-1* and *mnp1-2* were backcrossed with the wild type, respectively, and mutant plants were isolated from both F_2_ populations, followed by whole-genome resequencing at 20× coverage. Then, the resequencing data were analyzed using the bioinformatics tool Identification of Transposon Insertion Sites (ITIS) as previously described (Table S1) [47]. ITIS identified nine and seventy-one *Tnt1* insertions in the genomes of the *mnp1-1* and *mnp1-2* mutants, respectively. There were two *Tnt1* insertion sites on chromosome 7 that appeared to be nearby from the genomic sequence data of *mnp1-1* and *mnp1-2*; one was inserted into an intergenic region, and another was inserted into a genic region corresponding to the *Medtr7g011663* gene (annotated in A17 genome v4.0) (Figure 2A; Table S1). Then, PCR-based genotyping and sequencing analysis confirmed that the *mnp1-1* and *mnp1-2* mutants harbored *Tnt1* insertions in the fourth exon and the seventh exon of the candidate gene/*Medtr7g011663*, respectively (Figure 2B–D and Figure S2B). To determine whether the mutation of *Medtr7g011663* is responsible for the *mnp1* mutants’ phenotype, an additional mutant line with a predicted *Tnt1* insertion in *Medtr7g011663* locus was identified via BLAST searching of the public mutant database [45], and thus was designated as *mnp1-3.* The *mnp1-3* plants displayed a severely dwarfed phenotype similar to *mnp1* alleles when growing in the greenhouse (Figure 2C). PCR-based sequencing confirmed that there is indeed a *Tnt1* insertion in the sixth exon of *Medtr7g011663* in *mnp1-3* (Figure 2D and Figure S2B). Thus, we considered *Medtr7g011663* as the putative *MNP1* gene.

### 2.3. MNP1 Encodes a Putative CPS Protein in M. truncatula

**Figure 3:**
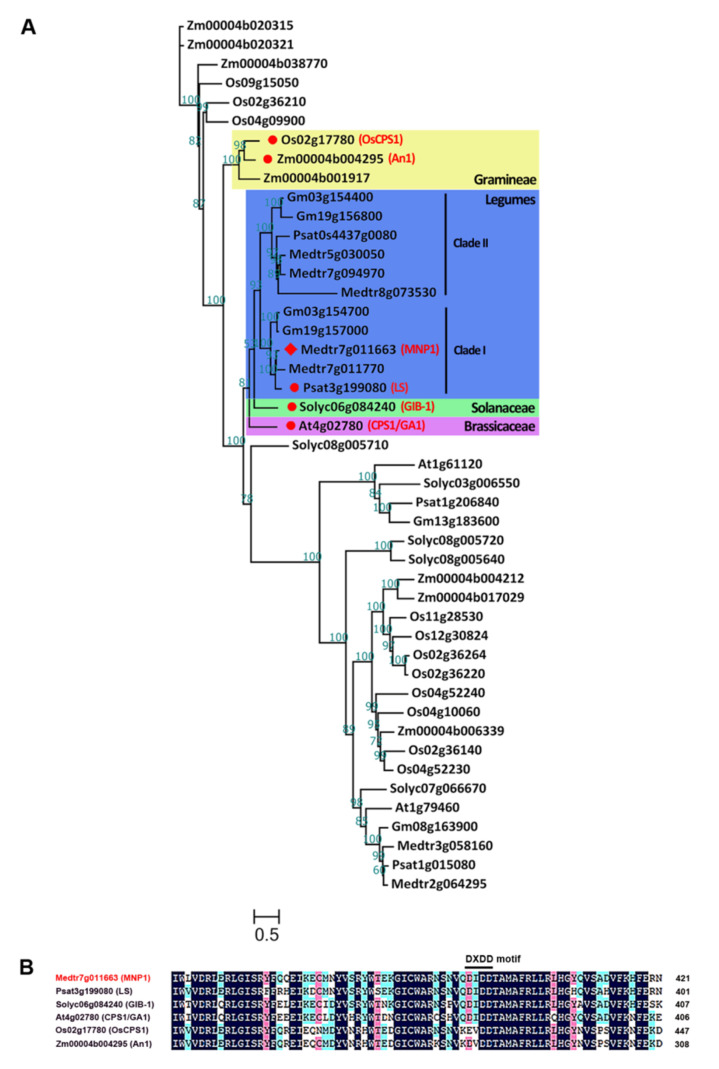
Phylogenetic analysis and sequences alignment of MNP1 and its closely related homologs. (**A**) Phylogenetic analysis of MNP1 and its homologs. Proteins from the species *Medicago truncatula* (Medtr), *Pisum sativum* (Psat), *Glycine max* (Gm), *Solanum lycopersicum* (Solyc), *Arabidopsis thaliana* (At), *Oryza sativa* (Os) and *Zea mays* (Zm). Bootstrap values are indicated upon the branches. Red rhombus indicates MNP1 protein and red circles indicate the reported CPS proteins. (**B**) The sequences alignment of MNP1 and the reported CPS proteins. The amino acid color indicates the homology of sequences between these species: black = 100%, pink ≥ 75% and blue ≥ 50%. The DXDD motifs in the sequences are indicated by the black line.

To figure out the type of protein encoded by *MNP1*, phylogenetic analysis of MNP1 and its homologous proteins from *M. truncatula* and related legume plants (pea and soybean), dicotyledonous model plants (*Arabidopsis* and tomato) and grasses (rice and maize) was performed. MNP1 protein was closely grouped with numerous homologs from legumes, and each selected legume species has at least two homologous copies. When compared to the reported homologous proteins, MNP1 showed the most homology to the pea LS and significant homology to the GIB-1 in tomatoes, CPS1/GA1 in *Arabidopsis*, OsCPS1 in rice and An1 in maize (Figure 3A), all of which are in the CPS family belonging to type-B cyclase and take part in the first step of GA biosynthesis [48,49,50,51,52]. The loss-of-function mutants of *ls*, *gib-1*, *cps1/ga1*, *Oscps1* and *an1* all show dwarfed phenotypes. In addition, the alignment of multiple amino acid sequences shows that MNP1 exhibits a high degree of amino acid sequence identities with these CPS proteins (Figure S3). Furthermore, there is an aspartate-rich motif DXDD near the N-terminal region of MNP1 (Figure 3B), which is conserved among type-B cyclase and important for the catalysis of the type-B cyclization reactions [52,53]. Taken together, we believe that MNP1 would be a conserved CPS protein involved in the GA biosynthesis pathway in *M. truncatula*.

### 2.4. Subcellular Localization of MNP1

The CPS1/GA1 has been reported to be localized on plastids in *Arabidopsis* with a chloroplast transit peptide (cTP) at its *N*-terminus [50]. Then, we carried out cTP prediction using the ChloroP program (http://www.cbs.dtu.dk/services/ChloroP) and found that the MNP1 is also highly predicted to have a cTP at its *N*-terminus, with a score of 0.591 (strong).

**Figure 4:**
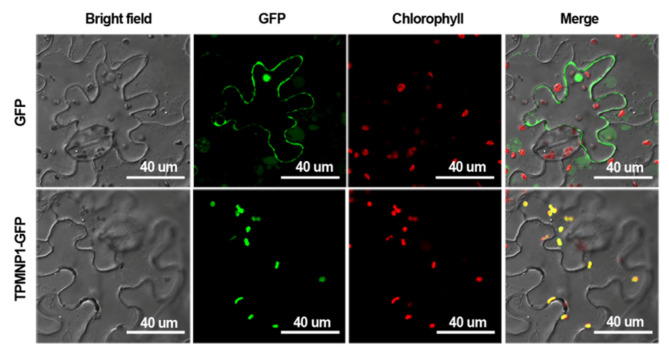
Subcellular localization of MNP1. According to ChloroP prediction, there is a chloroplast transit peptide (cTP) at the *N*-terminus of MNP1 protein, so a sequence encoding 100 amino acids containing cTP was used to generate *p35S::TPMNP1-GFP* constructs. Then, the constructs were transformed into tobacco (*Nicotiana benthamia*) leaf epidermal cells by *Agrobacterium*-mediated transformation. *p35S::GFP* was used as a positive control. Images were taken 36 h after transformation with dual GFP (green) and chlorophyll (red) channels. Scale bar = 40 um.

Based on the ChloroP prediction results, the sequence encoding the N-terminal truncation of 1–100 amino acids of MNP1 (TPMNP1) was used to generate *p35S::TPMNP1-GFP* constructs, which was then transiently expressed in epidermal cells of tobacco (*Nicotiana benthamia*). The green fluorescence signal of the fusion protein was observed in a chloroplast (Figure 4). This result was further confirmed by the subcellular localization analysis of the GFP fusion protein with full-length MNP1 (Figure S4). Thus, these data suggest that MNP1 may play the same role as *Arabidopsis* CPS1 in chloroplasts and participate in GA biosynthesis.

### 2.5. Genes of GA Biosynthesis Pathway Are Significantly Up-Regulated in mnp1-1

**Figure 5:**
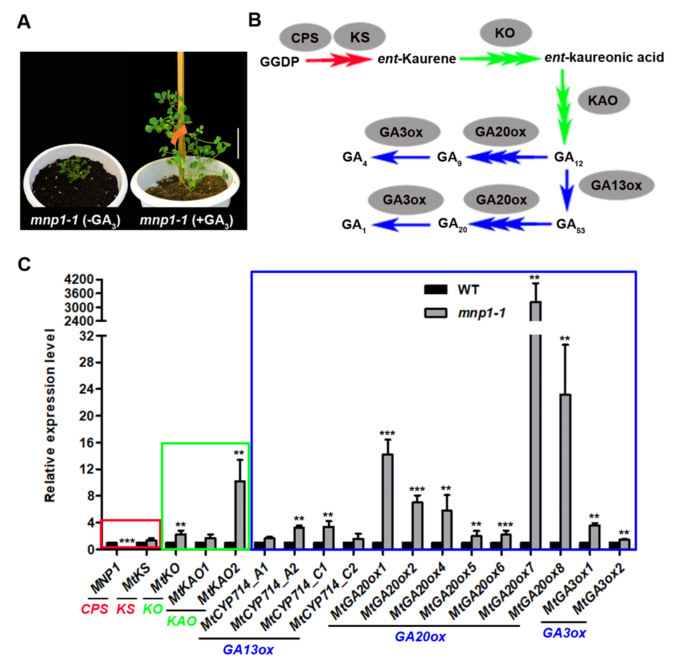
Expression analysis of GA biosynthesis genes in GA-sensitive mutant *mnp1-1*. (**A**) From left to right are *mnp1-1* without GA_3_ treatment and *mnp1-1* with 70 uM GA_3_ treatment. Scale bar = 4 cm. (**B**) GA biosynthesis pathway schematic diagram. The red, green and blue arrows represent the three stages of GA biosynthesis pathway. Gray ovals represent enzymes. (**C**) Relative expression levels of GA biosynthesis genes in the stem of WT and *mnp1-1*. The red, green and blue boxes represent the three stages of GA biosynthesis pathway as in (**B**). The significant difference was determined by unpaired two-sample *t*-test (** *p* < 0.01, *** *p* < 0.001).

Based on the above evidence, we conclude that *MNP1* is the putative gene encoding a CPS protein that participates in GA biosynthesis in *M. truncatula*. Therefore, exogenous GA_3_ was used to investigate whether *mnp1* is a GA-sensitive mutant. As expected, the plant height of *mnp1-1* sprayed with GA_3_ was significantly higher than that of the control group without GA treatment (Figure 5A). Besides, the blade size and petiole length of *mnp1-1* mutants were also significantly restored after GA treatment (Figure S5). Thus, it could be stated that the lack of GA leads to the dwarf phenotype of *mnp1.* The GA biosynthesis pathway involves many genes besides *CPS* (Figure 5B). According to the reference [54], we tested the expression level of the putative genes (Table S2) in the GA biosynthesis pathway of *M. truncatula* stem tissue. The results showed that most GA biosynthesis genes were highly upregulated in the *mnp1-1* mutant, while a small proportion of the genes (*MtKS*, *MtKAO1*, *MtCYP714_A1*, *MtCYP714_C2*) showed a low level of upregulation, with no significant difference. Among these upregulated genes, *MtGA20ox7* was most significant and was thousands of times higher than that of the wild type (Figure 5C), which is coincident with the earlier statement that GA20ox has an important role in GA homeostasis regulation in plants [20]. The upregulation of the genes that lie downstream of the GA biosynthesis pathway in *mnp1-1* implied a negative feedback response to the low GA content, and *MtGA20ox7* may play a key role in GA feedback regulation in *mnp1-1*.

### 2.6. MNP1 Could Partially Rescue the Phenotype of Arabidopsis cps1 Mutant

**Figure 6:**
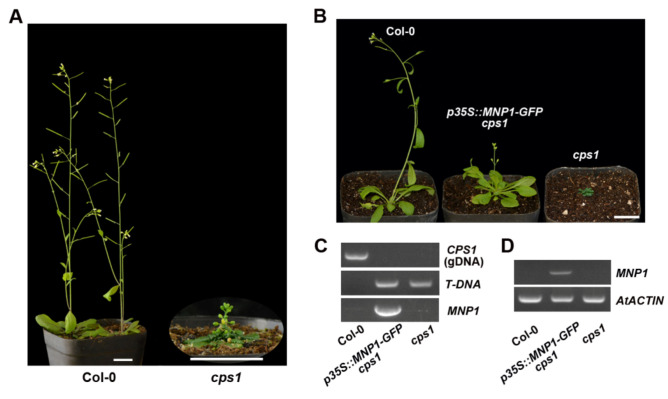
*MNP1* partially rescued the mini-plant phenotype of *Arabidopsis cps1* mutant. (**A**) The phenotype of *Arabidopsis* wild type (Col-0) and *cps1* mutant. The Col-0 and *cps1* mutant were 7 and 12 weeks old, respectively. Scale bar = 2 cm. (**B**) The *p35S::MNP1-GFP* transgenic plant of the *cps1* homozygous background partially restored the mini-plant phenotype of *cps1* mutant. The plants were 6 weeks old. Scale bar = 2 cm. (**C**) Genotyping of the transgenic plant. The homozygous *T-DNA* insertion in *CPS1/At4g02780* locus and *MNP1* coding sequence were detected in the transgenic plant. (**D**) RT-PCR amplification of *MNP1* from Col-0, the transgenic plant and *cps1* mutant. *AtACTIN* was used as an internal control.

The *cps1*/*ga1* mutant of *Arabidopsis* shows a severely dwarfed and sterile phenotype due to the loss of CPS function [55]. To examine the extent of the functional conservation between *M. truncatula* and *A. thaliana* CPS proteins, we obtained a homozygous *T-DNA* insertion mutant of *At4g02780* (SALK_109115) from the *Arabidopsis* Biological Resource Center (ABRC), namely the *cps1* mutant. The *cps1* mutant showed extremely dwarf as expected, and was able to produce inflorescences, but no fertile seeds (Figure 6A). Next, we introduced *p35S::MNP1-GFP* constructs into *cps1* heterozygotes by the floral dip method. Through resistance screening and PCR genotyping, we isolated the *p35S::MNP1-GFP* transgenic plants in the *cps1* homozygous background, and found that the size of transgenic plants was partially restored (Figure 6B,C). RT-PCR analysis confirmed the expression of *MNP1* gene in the transgenic plants (Figure 6D). These results indicated that *MNP1* could partially recover the mini-plant phenotype of the *cps1* mutant, suggesting that CPS has functional conservation between *M. truncatula* and *A. thaliana*.

## 3. Discussion

Although the genes encoding CPS have been identified in many species [48,49,50,51,52], the pea LS is the only CPS protein characterized from legumes in general before the present study, and *mnp1* appears to be the first dwarf mutant related to GA biosynthesis in *M. truncatula*. We found that the *mnp1* dwarf phenotype is caused by the decrease of the cell elongation and cell division in the stem. This result is consistent with the previously reported function of GA in promoting cell elongation and division. Cell elongation is regulated by cell wall-loosening protein expansin (EXP) and xyloglucan endo-transglycosylases (XET) which play a role in cell wall reconstruction. Some *XET* and *EXP* genes have been shown to be specifically upregulated by GA, which is believed to cause cell elongation in *Arabidopsis* and rice [56,57,58,59]. GA also promotes plant growth via upregulating the transcription levels of cell division-related genes including cell cycle genes *CYCA1;1* and *CDC2Os-3* in deepwater rice [60]. However, the underlying mechanism by which GA regulates the expression of these genes remains to be studied. The identification of *mnp1* provides a very good model to further study this mechanism in *M. truncatula*.

Focusing on the phylogenetic analysis of MNP1 and its homologous proteins, we found that CPS proteins belonging to legumes were grouped into two clades (clade I and II), and each clade was identified in all selected legumes, suggesting that a lineage-specific duplication of CPS genes may have occurred in legumes during the evolution process. There are just single copies from *Arabidopsis* and the tomato outgroup of the legume CPS proteins, while the CPS proteins of grasses gather together and are significantly separated from those of eudicots (Figure 3A). Consistent with this result, the conserved DXDD motif of CPS shows some degree of sequence divergence between monocots and eudicots (Figure 3B). In *M. truncatula*, MNP2/Medtr7g011770 appears to be a very close paralogue of MNP1/Medtr7g011663, because MNP1 and MNP2 are tightly clustered on chromosome 7 and shared high sequence identity. MNP1 and MNP2 belong to the legume CPS clade I, while in the legume CPS clade II, two members were found in the *M. truncatula* genome, namely MNP3/Medtr7g094970 and MNP4/Medtr5g030050. Since a highly conserved DXDD motif existed in all these four CPS proteins of *M. truncatula* (Figure S6), it will be interesting to explore the possibilities of functional redundancy and diversification between MNP1 and the rest members.

GA homeostasis is important for the regulation of many developmental processes and has been found to be maintained by feedback regulation of GA metabolism genes in a variety of plant species [61]. *GA20ox* and *GA3ox* are the main participants in the negative feedback regulation of GA. The expression of these two kinds of genes was upregulated in the GA biosynthesis deletion mutant [20]. In our study, we found that the expression of *MtGA20ox7* was significantly upregulated, up to thousands of times in *mnp1-1* compared with the wild type. Therefore, it seems that MtGA20ox7 may be a key member in the regulation of GA homeostasis in *M. truncatula.*

The *Arabidopsis cps1* mutants are male sterility caused by defective pollen development [55]. In *M. truncatula*, the expression of *MNP1* gene was also detected in stamens (Figure S7), suggesting that *MNP1* may play a potentially important role in stamen development. However, *mnp1* is fully fertile in *M. truncatula*, with its flower organ, pods and seeds being relatively smaller when compared with the wild type (Figure S8A–E). Pollen viability, tested by Alexander’s staining, indicated no significant difference between the wild type and *mnp1-1* as well (Figure S8C). It is a common phenomenon that GA deficiency leads to dwarfing and male sterility in various species, such as *Arabidopsis*, maize and tomato, but this scenario does not appear to be the case in legumes. Fertile pollens can be produced in all the reported dwarf mutants with GA deficiency in peas [43,51], and *mnp1* is similar to pea *ls* mutants, unlike *cps* mutants of other species. Given that a legume species usually contain multiple *CPS* genes, it can be argued that the mechanism of GA biosynthesis for plant height and pollen development in legumes may be conserved and distinct from that of other species. In terms of the dwarfed but fertile phenotypes of *mnp1* and *ls* mutants, the identification of *MNP1/LS* and other key genes involved in GA metabolism would be of great potential utility in legume breeding.

## 4. Materials and Methods

### 4.1. Plant Materials and Growth Conditions

*M. truncatula* ecotype R108 and *A. thaliana* ecotype Col-0 were used for this study. The *mnp1-1* (NF0500), *mnp1-2* (NF13564) and *mnp1-3* (NF10616) mutants (all in ecotype R108 background) were isolated from the *Tnt1* retrotransposon-tagged mutant collection of *M. truncatula* as previously reported [45]. Among them, the *mnp1-3* mutants were screened from the *Tnt1* population by a reverse genetic approach. Seeds of *Arabidopsis cps1* mutant (SALK_109115) were purchased from the *Arabidopsis* Biological Resource Center (ABRC). The *p35S::MNP1-GFP* transgenic plants were generated in *cps1* background.

*Arabidopsis* GA-deficient mutant *cps1* cannot germinate in the soil. For this reason, *Arabidopsis* plants need to be grown on solid 1/2 MS medium for approximately 2 weeks and then be transplanted into the soil. All plants (*M. truncatula* and *A. thaliana*) were grown under the following greenhouse conditions: 16 h day/8 h night cycle, 150 uE/m^2^/s light intensity, 22 °C day/18 °C night temperature and 70% humidity.

### 4.2. Statistical Analysis of Cell Length and Number

For the measurement of the internode length, twenty individual plants of both the wild type and *mnp1-1* genotypes were grown simultaneously in the same greenhouse, and the third internode beneath the shoot apex of each plant (2-month-old) was collected and considered as an independent biological sample. Thus, a total of twenty internodes were processed to calculate the average length. Then, three internodes of each genotype were randomly selected from the above twenty samples, and were submerged in fixative solution (5% formaldehyde, 5% acetic acid and 50% ethanol) for over 12 h at room temperature. Subsequently, the samples were dehydrated in a graded ethanol series (50%, 70%, 90%, 95%, 100%), critical-point dried in liquid CO_2_ and sputter-coated with gold. The three dried internodes for each genotype were individually examined using scanning electron microscopy (SEM) by an EVO LS10 (Zeiss, Oberkochen, Germany) at an accelerating voltage of 5 kV. Therefore, three SEM images of the third internode were obtained for each genotype (wild type and *mnp1-1*).

For the measurement of epidermal cell length of the internode, 20 cells were randomly selected from the SEM images (6–7 cells per image) for both the wild type and *mnp1-1* genotypes, and the lengths were measured by ImageJ.

The cell number was calculated from the ratio of the average internode length (that was evaluated from a total of 20 internodes) to the average cell length (that was evaluated from 20 cells of three biological replicates).

All above experiments were repeated twice independently with similar results.

### 4.3. Molecular Cloning of MNP1

The molecular cloning of the *MNP1* gene referred to the method reported previously [62]. We screened the *Tnt1* retrotransposon insertion mutant collection of *M. truncatula* (ecotype R108) and isolated two *mnp1* alleles (*mnp1-1*, NF0500 and *mnp1-2*, NF13564) with severely dwarfed phenotypes. Then, these two *mnp1* alleles were backcrossed with the wild type to purify the genetic background for reducing incoherent *Tnt1* insertions, and *mnp1-1* and *mnp1-2* F_2_ segregation populations were generated, respectively. Equal amounts of leaf material were harvested from 12 independent mutant individuals of each population to make two mixed samples. The genomic DNA of the mixed samples was extracted using the Plant Genomic DNA Kit (Tiangen, Beijing, China). Whole-genome resequencing was carried out at 20× coverage. Then, the data of whole-genome resequencing were analyzed by a novel bioinformatics tool, Identification of Transposon Insertion Sites (ITIS) to identify all *Tnt1* insertion sites in the genome [47]. The common *Tnt1* insertion sites in *mnp1-1* and *mnp1-2* genomes were found in *Medtr7g011663* locus (annotated in A17 genome v4.0). Subsequently, PCR experiments using *mnp1-1* and *mnp1-2* genomic DNA as templates were performed to verify the insertion of *Tnt1* in *Medtr7g011663*. An additional allele *mnp1-3* (NF10616) was screened from the *Tnt1* population by a reverse genetics approach, which also displayed a mini-plant phenotype. Genomic PCR analysis confirmed that the *mnp1-3* mutant does carry a *Tnt1* insertion in *Medtr7g011663.* Thus, *Medtr7g011663* was regarded as the putative *MNP1* gene. The analysis data of ITIS and the primers used for PCR are shown in Tables S1 and S3, respectively.

### 4.4. Phylogenetic Analysis and Sequences Alignment

The sequences of MNP1 homologs were identified through BLAST from Phytozome (https://phytozome.jgi.doe.gov/pz/portal.html), URGI (https://urgi.versailles.inra.fr/Species/Pisum) and maizeGDB (https://maizegdb.org/) in the protein databases of *Medicago truncatula, Glycine max, Solanum lycopersicum, Arabidopsis thaliana*, *Oryza sativa*, *Pisum sativum* and *Zea mays*. Multiple amino acid sequences were aligned by ClustalX2 (v2.1) at default parameters and beautified by DNAMAN V6. The phylogenetic tree was performed by the maximum likelihood method with IQTREE v1.6.10 as previously reported [63]. The JTT + F + G4 model was selected as suggested by the IQTREE model test tool (BIC criterion) with 1000 ultrafast bootstrap replicates and 5000 iterations.

### 4.5. Exogenous GA_3_ Application Method

Bioactive GA_3_ (Genview, Lot: 5209010140) was dissolved in ethanol (0.1 M) and diluted with water before being applied [64]. About 600 mL of 70 uM bioactive GA_3_ working solution was sprayed to a total of twelve *mnp1-1* mutant plant one time. The first spray was applied at 10-day-old seedlings after sowing, and the later sprays performed once a week for two months in total. An equivalent group (*n* = 12) of *mnp1-1* mutant plants was treated similarly with a solution without GA_3_ at each same time. All *mnp1-1* mutants with the treatments (+GA_3_ and −GA_3_) were grown simultaneously in the same greenhouse. Experiments were repeated twice independently with similar results.

### 4.6. RNA Extraction, RT-PCR and Quantitative RT-PCR (qRT-PCR)

*Medicago* stem tissues and *Arabidopsis* rosette leaves for RNA extraction were harvested from 7-week-old and 6-week-old plants, respectively. Total RNA was isolated using TransZol (TransGen, Beijing, China) according to the manufacturer’s protocol and then was reverse transcribed into cDNA by HiScript^®^ II 1st Strand cDNA Synthesis Kit (Vazyme, Nanjing, China). The resulting cDNAs were used as templates for RT-PCR and qRT-PCR. *AtACTIN* (*Actin2*/*At3g18780*) was used as an internal control for *Arabidopsis* RT-PCR. qRT-PCR was performed using 2 × T5 Fast qPCR mix (SYBR Green I) (TsingKe, Beijing, China) on the Roche Light Cycler 480II real-time PCR machine (95 °C, 1 min; 95 °C, 10 s, 60 °C, 10 s, 72 °C, 15 s, 40 cycles). *MtACTIN* (*Medtr3g095530*) was used as an internal control for *Medicago* qPCR. Three independent biological replicates were used for RNA extraction and subsequent cDNA synthesis. All samples were selected randomly under the same greenhouse conditions. Three technical replicates for each biological replicate were used in qRT-PCR analysis. The genes involved in this study and the primers used for qPCR are listed in Tables S2 and S3, respectively.

### 4.7. Plasmid Construction

Coding sequences of target genes were isolated by RT-PCR from wild type root tissue of seedlings (3 weeks old). For subcellular localization experiments, the coding sequences of the *N*-terminus of MNP1 (100-amino acid, TPMNP1) and the full-length coding sequence of MNP1 were inserted into the *pCAMBIA3301MP* vector between NcoI and AvrII site via the ClonExpress II One Step Cloning Kit (Vazyme) to generate *p35S::TPMNP1-GFP* and *p35S::MNP1-GFP* constructs, respectively. The *p35S:: MNP1-GFP* constructs were also used for plant transformation. The primers used for plasmid construction are listed in Table S3.

### 4.8. Subcellular Localization

The constructs, *p35S::TPMNP1-GFP* and *p35S::GFP*, were introduced into *Agrobacterium tumefaciens* EHA105 strain, and then they were transiently expressed in tobacco (*Nicotiana benthamia*) leaves by *Agrobacterium*-mediated transformation [65]. The *p35S::GFP* constructs were served as a positive control. The TPMNP1-GFP fusion protein was examined using a confocal laser scanning microscope (FV1000; Olympus, Japan). This experiment was repeated three times independently with similar results.

### 4.9. Plant Transformation

The *p35S::MNP1-GFP* constructs were introduced into *Agrobacterium tumefaciens* EHA105 strain, which was subsequently used to transform *cps1* heterozygotes by *Agrobacterium*-mediated transformation using the floral dip method [66]. Through resistance screening with 20 mg/L Basta (BBI Life Sciences, Lot: C707BA0017) and the subsequent PCR genotyping, the *p35S::MNP1-GFP* transgenic lines in the *cps1* homozygous background were isolated. The primers used for PCR are shown in Table S3.

### 4.10. Alexander’s Staining

Mature pollens were stained with Alexander’s staining solution as previously described [67]. Mature anthers of WT and *mnp1-1* from the same developmental stage were immersed directly in a drop of staining solution, covered with a coverslip respectively, and then kept them in an oven at 50 °C for 1 h. Next, a microscopic examination was conducted via a fluorescence microscope (Olympus BX63) using the bright field channel. The fertile pollen would be stained red to deep red, while aborted pollen would be green. Experiments were repeated more than three times independently.

## Supplementary Materials

Supplementary materials can be found at https://www.mdpi.com/1422-0067/21/14/4968/s1.

## References

[1] Spielmeyer W., Ellis M.H., Chandler P.M. (2002). Semidwarf (sd-1), “green revolution” rice, contains a defective gibberellin 20-oxidase gene. Proc. Natl. Acad. Sci. USA.

[2] Peng J., Richards D.E., Hartley N.M., Murphy G.P., Devos K.M., Flintham J.E., Beales J., Fish L.J., Worland A.J., Pelica F. (1999). ‘Green revolution’ genes encode mutant gibberellin response modulators. Nature.

[3] Nelissen H., Rymen B., Jikumaru Y., Demuynck K., Van Lijsebettens M., Kamiya Y., Inze D., Beemster G.T. (2012). A local maximum in gibberellin levels regulates maize leaf growth by spatial control of cell division. Curr. Biol..

[4] Cao D., Cheng H., Wu W., Soo H.M., Peng J. (2006). Gibberellin mobilizes distinct DELLA-dependent transcriptomes to regulate seed germination and floral development in Arabidopsis. Plant Physiol..

[5] King R.W., Moritz T., Evans L.T., Junttila O., Herlt A.J. (2001). Long-day induction of flowering in Lolium temulentum involves sequential increases in specific gibberellins at the shoot apex. Plant Physiol..

[6] Lo S.F., Yang S.Y., Chen K.T., Hsing Y.I., Zeevaart J.A., Chen L.J., Yu S.M. (2008). A novel class of gibberellin 2-oxidases control semidwarfism, tillering, and root development in rice. Plant Cell.

[7] Davidson S.E., Elliott R.C., Helliwell C.A., Poole A.T., Reid J.B. (2003). The pea gene NA encodes ent-kaurenoic acid oxidase. Plant Physiol..

[8] Yamaguchi S. (2008). Gibberellin metabolism and its regulation. Annu. Rev. Plant Biol..

[9] Magome H., Nomura T., Hanada A., Takeda-Kamiya N., Ohnishi T., Shinma Y., Katsumata T., Kawaide H., Kamiya Y., Yamaguchi S. (2013). CYP714B1 and CYP714B2 encode gibberellin 13-oxidases that reduce gibberellin activity in rice. Proc. Natl. Acad. Sci. USA.

[10] Duncan J.D., West C.A. (1981). Properties of Kaurene Synthetase from Marah macrocarpus Endosperm: Evidence for the Participation of Separate but Interacting Enzymes. Plant Physiol..

[11] Helliwell C.A., Sullivan J.A., Mould R.M., Gray J.C., Peacock W.J., Dennis E.S. (2001). A plastid envelope location of Arabidopsis ent-kaurene oxidase links the plastid and endoplasmic reticulum steps of the gibberellin biosynthesis pathway. Plant J..

[12] Helliwell C.A., Sheldon C.C., Olive M.R., Walker A.R., Zeevaart J.A., Peacock W.J., Dennis E.S. (1998). Cloning of the Arabidopsis ent-kaurene oxidase gene GA3. Proc. Natl. Acad. Sci. USA.

[13] Helliwell C.A., Chandler P.M., Poole A., Dennis E.S., Peacock W.J. (2001). The CYP88A cytochrome P450, ent-kaurenoic acid oxidase, catalyzes three steps of the gibberellin biosynthesis pathway. Proc. Natl. Acad. Sci. USA.

[14] Xu Y.L., Li L., Wu K., Peeters A.J., Gage D.A., Zeevaart J.A. (1995). The GA5 locus of Arabidopsis thaliana encodes a multifunctional gibberellin 20-oxidase: Molecular cloning and functional expression. Proc. Natl. Acad. Sci. USA.

[15] Lange T., Hedden P., Graebe J.E. (1994). Expression cloning of a gibberellin 20-oxidase, a multifunctional enzyme involved in gibberellin biosynthesis. Proc. Natl. Acad. Sci. USA.

[16] Chiang H.H., Hwang I., Goodman H.M. (1995). Isolation of the Arabidopsis GA4 locus. Plant Cell.

[17] Israelsson M., Mellerowicz E., Chono M., Gullberg J., Moritz T. (2004). Cloning and overproduction of gibberellin 3-oxidase in hybrid aspen trees. Effects on gibberellin homeostasis and development. Plant Physiol..

[18] Regnault T., Daviere J.M., Heintz D., Lange T., Achard P. (2014). The gibberellin biosynthetic genes AtKAO1 and AtKAO2 have overlapping roles throughout Arabidopsis development. Plant J..

[19] Rieu I., Ruiz-Rivero O., Fernandez-Garcia N., Griffiths J., Powers S.J., Gong F., Linhartova T., Eriksson S., Nilsson O., Thomas S.G. (2008). The gibberellin biosynthetic genes AtGA20ox1 and AtGA20ox2 act, partially redundantly, to promote growth and development throughout the Arabidopsis life cycle. Plant J..

[20] Hedden P., Phillips A.L. (2000). Gibberellin metabolism: New insights revealed by the genes. Trends Plant Sci..

[21] Griffiths J., Murase K., Rieu I., Zentella R., Zhang Z.L., Powers S.J., Gong F., Phillips A.L., Hedden P., Sun T.P. (2006). Genetic characterization and functional analysis of the GID1 gibberellin receptors in Arabidopsis. Plant Cell.

[22] Willige B.C., Ghosh S., Nill C., Zourelidou M., Dohmann E.M., Maier A., Schwechheimer C. (2007). The DELLA domain of GA INSENSITIVE mediates the interaction with the GA INSENSITIVE DWARF1A gibberellin receptor of Arabidopsis. Plant Cell.

[23] Varbanova M., Yamaguchi S., Yang Y., McKelvey K., Hanada A., Borochov R., Yu F., Jikumaru Y., Ross J., Cortes D. (2007). Methylation of gibberellins by Arabidopsis GAMT1 and GAMT2. Plant Cell.

[24] Zhu Y., Nomura T., Xu Y., Zhang Y., Peng Y., Mao B., Hanada A., Zhou H., Wang R., Li P. (2006). ELONGATED UPPERMOST INTERNODE encodes a cytochrome P450 monooxygenase that epoxidizes gibberellins in a novel deactivation reaction in rice. Plant Cell.

[25] Wuddineh W.A., Mazarei M., Zhang J., Poovaiah C.R., Mann D.G., Ziebell A., Sykes R.W., Davis M.F., Udvardi M.K., Stewart C.N. (2015). Identification and overexpression of gibberellin 2-oxidase (GA2ox) in switchgrass (Panicum virgatum L.) for improved plant architecture and reduced biomass recalcitrance. Plant Biotechnol. J..

[26] Szekeres M., Nemeth K., Koncz-Kalman Z., Mathur J., Kauschmann A., Altmann T., Redei G.P., Nagy F., Schell J., Koncz C. (1996). Brassinosteroids rescue the deficiency of CYP90, a cytochrome P450, controlling cell elongation and de-etiolation in Arabidopsis. Cell.

[27] Li J., Wen J., Lease K.A., Doke J.T., Tax F.E., Walker J.C. (2002). BAK1, an Arabidopsis LRR receptor-like protein kinase, interacts with BRI1 and modulates brassinosteroid signaling. Cell.

[28] Zhou F., Lin Q., Zhu L., Ren Y., Zhou K., Shabek N., Wu F., Mao H., Dong W., Gan L. (2013). D14-SCF(D3)-dependent degradation of D53 regulates strigolactone signalling. Nature.

[29] Lin H., Wang R., Qian Q., Yan M., Meng X., Fu Z., Yan C., Jiang B., Su Z., Li J. (2009). DWARF27, an iron-containing protein required for the biosynthesis of strigolactones, regulates rice tiller bud outgrowth. Plant Cell.

[30] Yokota T. (1997). The structure, biosynthesis and function of brassinosteroids. Trend Plant Sci..

[31] Choe S., Dilkes B.P., Gregory B.D., Ross A.S., Yuan H., Noguchi T., Fujioka S., Takatsuto S., Tanaka A., Yoshida S. (1999). The Arabidopsis dwarf1 mutant is defective in the conversion of 24-methylenecholesterol to campesterol in brassinosteroid biosynthesis. Plant Physiol..

[32] Arite T., Iwata H., Ohshima K., Maekawa M., Nakajima M., Kojima M., Sakakibara H., Kyozuka J. (2007). DWARF10, an RMS1/MAX4/DAD1 ortholog, controls lateral bud outgrowth in rice. Plant J..

[33] Clouse S.D. (2011). Brassinosteroid signal transduction: From receptor kinase activation to transcriptional networks regulating plant development. Plant Cell.

[34] Li J., Nam K.H., Vafeados D., Chory J. (2001). BIN2, a new brassinosteroid-insensitive locus in Arabidopsis. Plant Physiol..

[35] Jiang L., Liu X., Xiong G., Liu H., Chen F., Wang L., Meng X., Liu G., Yu H., Yuan Y. (2013). DWARF 53 acts as a repressor of strigolactone signalling in rice. Nature.

[36] Ishikawa S., Maekawa M., Arite T., Onishi K., Takamure I., Kyozuka J. (2005). Suppression of tiller bud activity in tillering dwarf mutants of rice. Plant Cell Physiol..

[37] Maphosa Y., Jideani V.A., Chavarri M. (2017). The Role of Legumes in Human Nutrition. Functional Food.

[38] Li Z.F., Guo Y., Ou L., Hong H., Wang J., Liu Z.X., Guo B., Zhang L., Qiu L. (2018). Identification of the dwarf gene GmDW1 in soybean (Glycine max L.) by combining mapping-by-sequencing and linkage analysis. TAG Theor. Appl. Genet..

[39] Reid J.B., Ross J.J. (1993). A Mutant-Based Approach, Using Pisum sativum, to Understanding Plant Growth. Int. J. Plant Sci..

[40] Yaxley J.R., Ross J.J., Sherriff L.J., Reid J.B. (2001). Gibberellin biosynthesis mutations and root development in pea. Plant Physiol..

[41] Tang H., Krishnakumar V., Bidwell S., Rosen B., Chan A., Zhou S., Gentzbittel L., Childs K.L., Yandell M., Gundlach H. (2014). An improved genome release (version Mt4.0) for the model legume Medicago truncatula. BMC Genom..

[42] Pecrix Y., Staton S.E., Sallet E., Lelandais-Brière C., Moreau S., Carrère S., Blein T., Jardinaud M.F., Latrasse D., Zouine M. (2018). Whole-genome landscape of Medicago truncatula symbiotic genes. Nat. Plants.

[43] Swain S.M., Ross J.J., Reid J.B., Kamiya Y. (1995). Gibberellins and pea seed development: Expression of the lh^i, ls and le^5839 mutations. Planta.

[44] Burks D., Azad R., Wen J., Dickstein R. (2018). The Medicago truncatula Genome: Genomic Data Availability. Methods Mol. Biol..

[45] Tadege M., Wen J., He J., Tu H., Kwak Y., Eschstruth A., Cayrel A., Endre G., Zhao P.X., Chabaud M. (2008). Large-scale insertional mutagenesis using the Tnt1 retrotransposon in the model legume Medicago truncatula. Plant J..

[46] Hacham Y., Holland N., Butterfield C., Ubeda-Tomas S., Bennett M.J., Chory J., Savaldi-Goldstein S. (2011). Brassinosteroid perception in the epidermis controls root meristem size. Development.

[47] Jiang C., Chen C., Huang Z., Liu R., Verdier J. (2015). ITIS, a bioinformatics tool for accurate identification of transposon insertion sites using next-generation sequencing data. BMC Bioinform..

[48] Bensen R.J., Johal G.S., Crane V.C., Tossberg J.T., Schnable P.S., Meeley R.B., Briggs S.P. (1995). Cloning and characterization of the maize An1 gene. Plant Cell.

[49] Jacobsen S.E., Olszewski N.E. (1991). Characterization of the Arrest in Anther Development Associated with Gibberellin Deficiency of the gib-1 Mutant of Tomato. Plant Physiol..

[50] Sun T.P., Kamiya Y. (1994). The Arabidopsis GA1 locus encodes the cyclase ent-kaurene synthetase A of gibberellin biosynthesis. Plant Cell.

[51] Ait-Ali T., Swain S.M., Reid J.B., Sun T., Kamiya Y. (1997). The LS locus of pea encodes the gibberellin biosynthesis enzyme ent-kaurene synthase A. Plant J..

[52] Otomo K., Kenmoku H., Oikawa H., Konig W.A., Toshima H., Mitsuhashi W., Yamane H., Sassa T., Toyomasu T. (2004). Biological functions of ent- and syn-copalyl diphosphate synthases in rice: Key enzymes for the branch point of gibberellin and phytoalexin biosynthesis. Plant J..

[53] Prisic S., Xu J., Coates R.M., Peters R.J. (2007). Probing the Role of the DXDD Motif in Class II Diterpene Cyclases. ChemBioChem.

[54] Igielski R., Kepczynska E. (2017). Gene expression and metabolite profiling of gibberellin biosynthesis during induction of somatic embryogenesis in Medicago truncatula Gaertn. PLoS ONE.

[55] Cheng H., Qin L., Lee S., Fu X., Richards D.E., Cao D., Luo D., Harberd N.P., Peng J. (2004). Gibberellin regulates Arabidopsis floral development via suppression of DELLA protein function. Development.

[56] Xu W., Purugganan M.M., Polisensky D.H., Antosiewicz D.M., Fry S.C., Braam J. (1995). Arabidopsis TCH4, regulated by hormones and the environment, encodes a xyloglucan endotransglycosylase. Plant Cell.

[57] Uozu S., Tanaka-Ueguchi M., Kitano H., Hattori K., Matsuoka M. (2000). Characterization of XET-related genes of rice. Plant Physiol..

[58] Lee Y., Kende H. (2001). Expression of beta-expansins is correlated with internodal elongation in deepwater rice. Plant Physiol..

[59] Lee Y., Kende H. (2002). Expression of alpha-expansin and expansin-like genes in deepwater rice. Plant Physiol..

[60] Fabian T., Lorbiecke R., Umeda M., Sauter M. (2000). The cell cycle genes cycA1;1 and cdc2Os-3 are coordinately regulated by gibberellin in planta. Planta.

[61] Gallego-Giraldo L., Ubeda-Tomas S., Gisbert C., Garcia-Martinez J.L., Moritz T., Lopez-Diaz I. (2008). Gibberellin homeostasis in tobacco is regulated by gibberellin metabolism genes with different gibberellin sensitivity. Plant Cell Physiol..

[62] Zhao B., He L., Jiang C., Liu Y., He H., Bai Q., Zhou S., Zheng X., Wen J., Mysore K.S. (2020). Lateral Leaflet Suppression 1 (LLS1), encoding the MtYUCCA1 protein, regulates lateral leaflet development in Medicago truncatula. New Phytol..

[63] He L., Liu Y., He H., Liu Y., Qi J., Zhang X., Li Y., Mao Y., Zhou S., Zheng X. (2020). A molecular framework underlying the compound leaf pattern of Medicago truncatula. Nat. Plants.

[64] Dalmadi A., Kalo P., Jakab J., Saskoi A., Petrovics T., Deak G., Kiss G.B. (2008). Dwarf plants of diploid Medicago sativa carry a mutation in the gibberellin 3-beta-hydroxylase gene. Plant Cell Rep..

[65] Kapila J., Rycke R.D., Montagu M.V., Angenon G. (1997). An Agrobacterium-mediated transient gene expression system for intact leaves. Plant Sci..

[66] Clough S.J., Bent A.F. (1998). Floral dip: A simplified method for Agrobacterium-mediated transformation of Arabidopsis thaliana. Plant J..

[67] Alexander M.P. (1969). Differential staining of aborted and nonaborted pollen. Stain Technol..

