# Supplementary material for: Cloning and Functional Analysis of Dwarf Gene Mini Plant 1 (MNP1) in Medicago truncatula
Source: Int J Mol Sci. 2020 Jul 14;21(14):4968. doi: 10.3390/ijms21144968 (PMC7404263; doi:10.3390/ijms21144968)
Supplement: Supplementary file 1 [file ijms-21-04968-s001.zip › ijms-837134-supplementary-final/Supplementary Materials.docx]

**Cloning and Functional Analysis of Dwarf Gene *Mini Plant 1* (*MNP1*) in *Medicago truncatula***

**
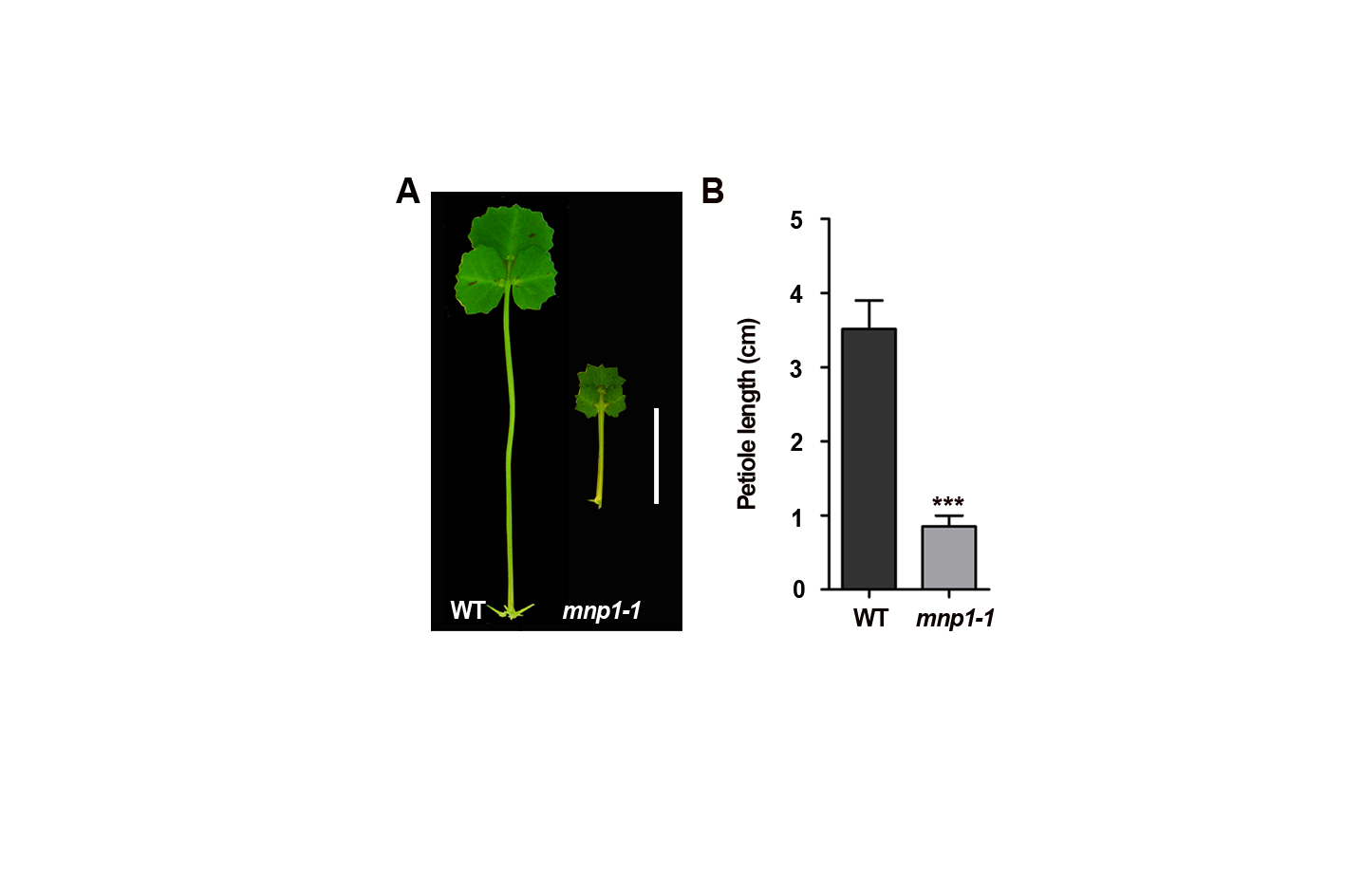
**

**Figure S1.** The petiole of *mnp1-1* is shorter than that of the wild type (WT). **(A)** The first compound leaf of WT and *mnp1-1* seedlings. Scale bar =1 cm. **(B)** Comparisons of petiole length of the first compound leaf between WT and *mnp1-1* seedlings*.* Values are means ± *SD* (n =20). Two-sample *t*-test, ****P*<0.001.

**
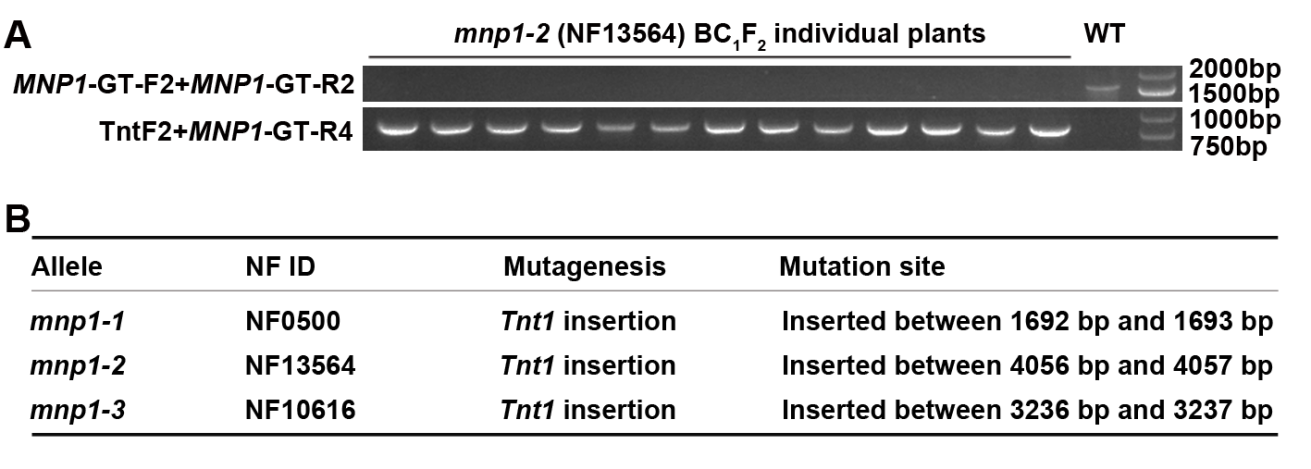
**

**Figure S2.** Cosegregation analysis and mutation sites of *mnp1* alleles. **(A)** Thirteen independent mutants from the *mnp1-2* BC_1_F_2_ population were used for cosegregation analysis, and they are all homozygous for *Tnt1* insertion in *MNP1*/*Medtr7g011663* locus. The primer pair *MNP1*-GT-F2/R2 was used for detecting *MNP1* genomic fragment, and the primer pair TntF2/*MNP1*-GT-R4 was used for verifying the existence of *Tnt1* insertion within the *MNP1*/*Medtr7g011663* genomic region. **(B)** *Tnt1* was inserted at position 1692 bp, 4056 bp and 3236 bp downstream from the translational start of *MNP1*/*Medtr7g011663* in *mnp1-1*, *mnp1-2* and *mnp1-3* mutants, respectively.

**
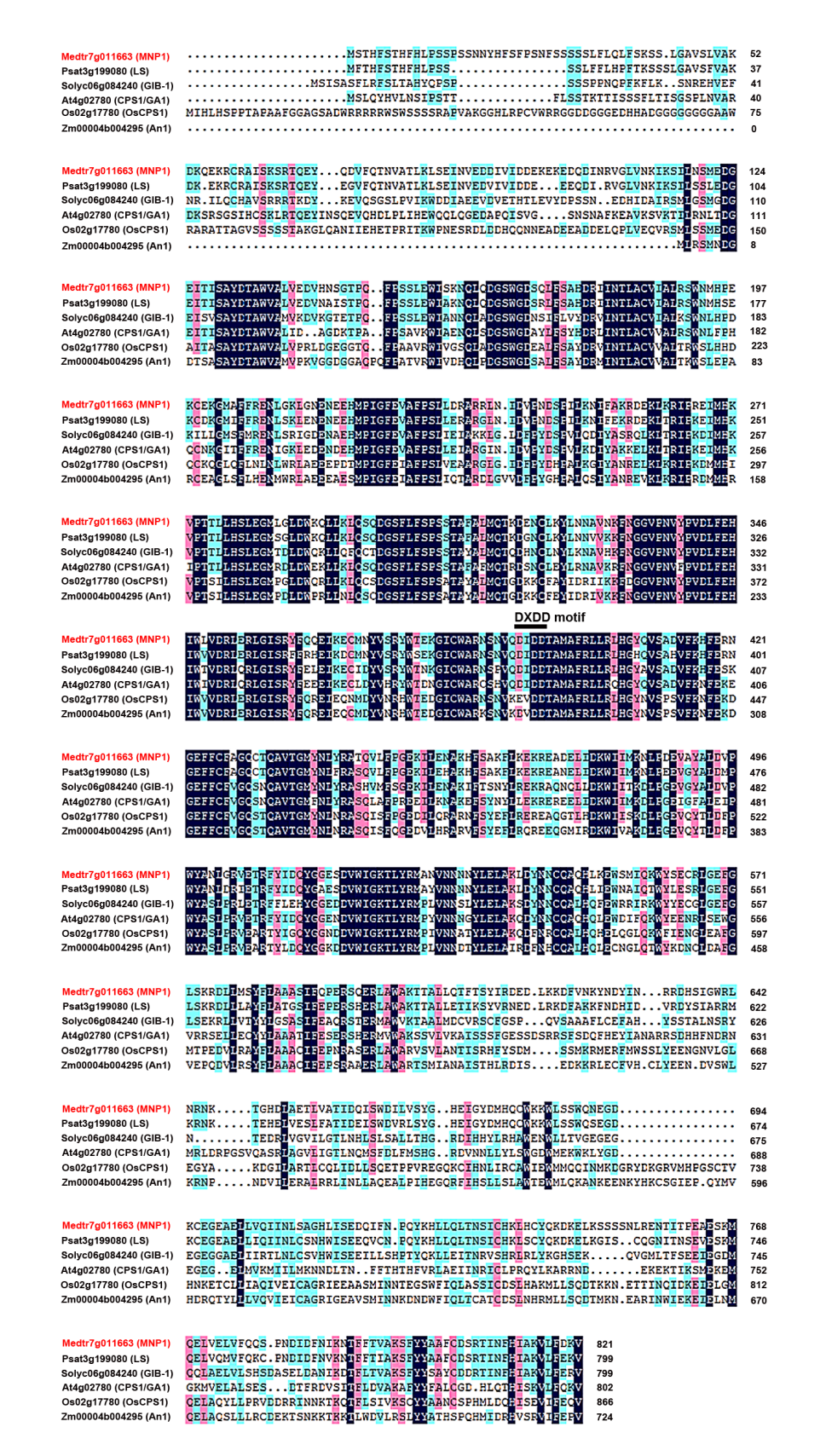
**

**Figure S3.** The sequences alignment of MNP1 and the reported CPS proteins. The amino acid color indicates the homology of sequences between different species: black =100%, pink≧75% and blue≧50%. The DXDD motifs in the sequences are indicated by the black line.


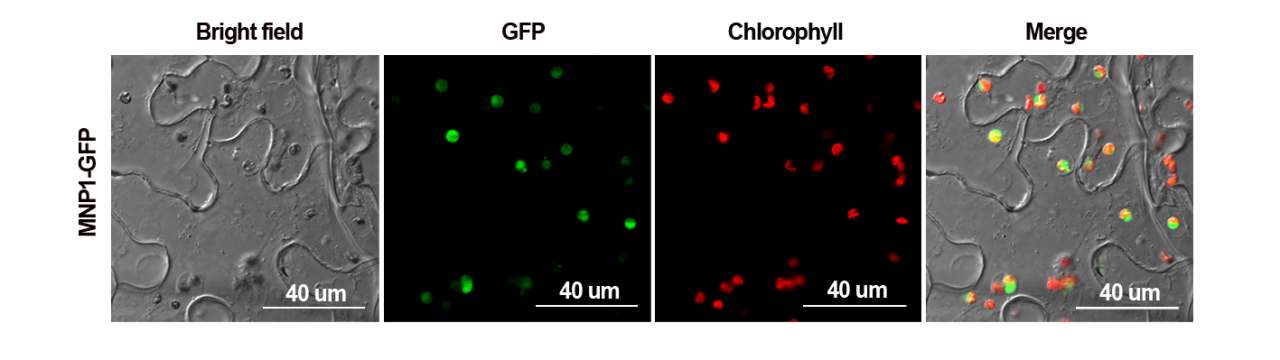


**Figure S4.** Subcellular localization of MNP1. Subcellular localization of the MNP1-GFP fusion protein in tobacco (*Nicotiana benthamia*) leaf epidermal cells. Images were taken 36 h after transformation with dual GFP (green) and chlorophyll (red) channels. Scale bar =40 um.

**
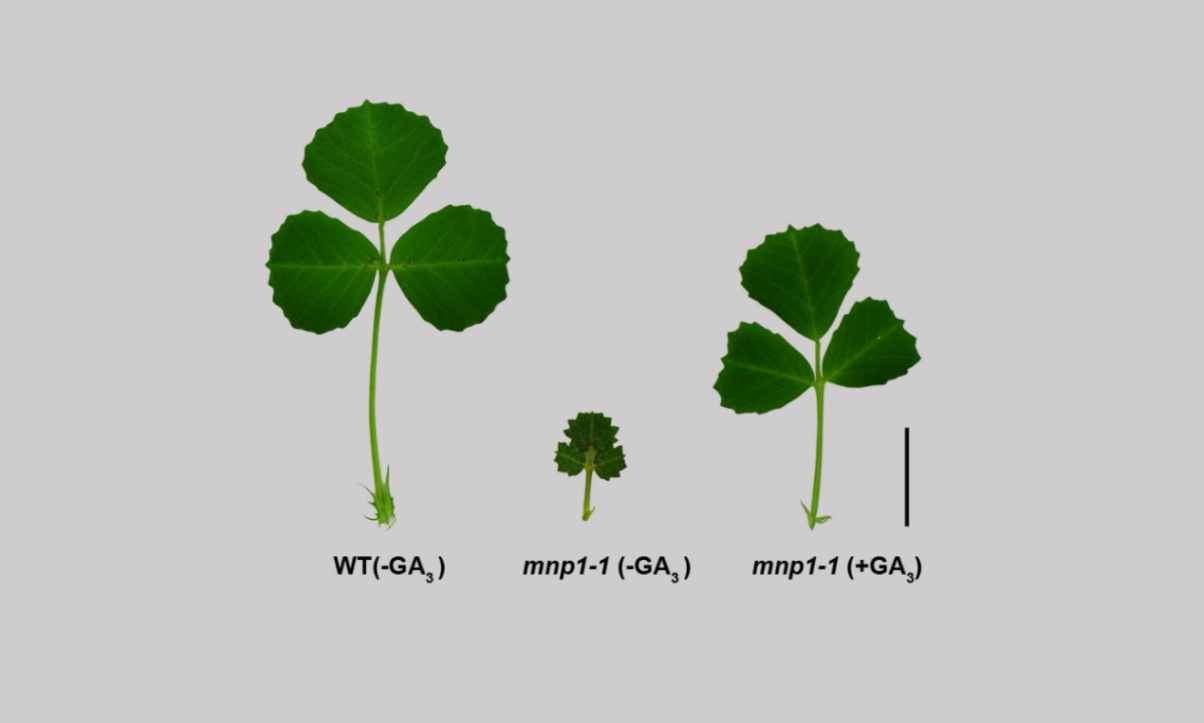
**

**Figure S5.** The leaf phenotype of *mnp1-1* was partially restored after GA_3_ treatment. From left to right are compound leaf closest to shoot apex of WT seedling without GA_3_ treatment, *mnp1-1* seedling without GA_3_ treatment and *mnp1-1* seedling with 70 uM GA_3_ treatment, respectively. Scale bar =1 cm.


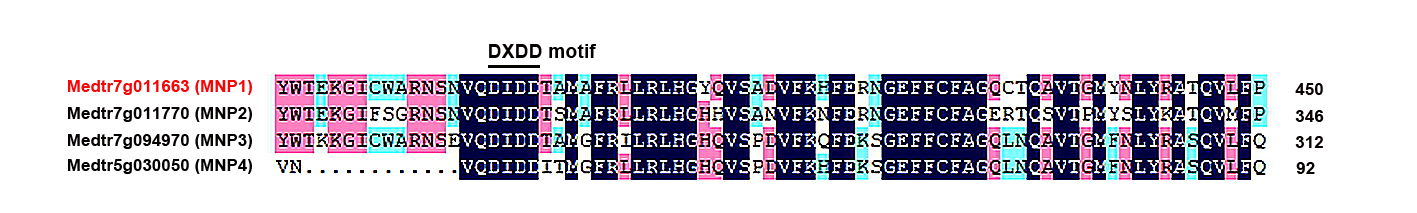


**Figure S6.** The sequences alignment of MNP1/Medtr7g011663 and its closely related homologs in *M. truncatula.* The amino acid color indicates the homology of sequences: black =100%, pink≧75% and blue≧50%. The DXDD motifs in the sequences are indicated by the black line.


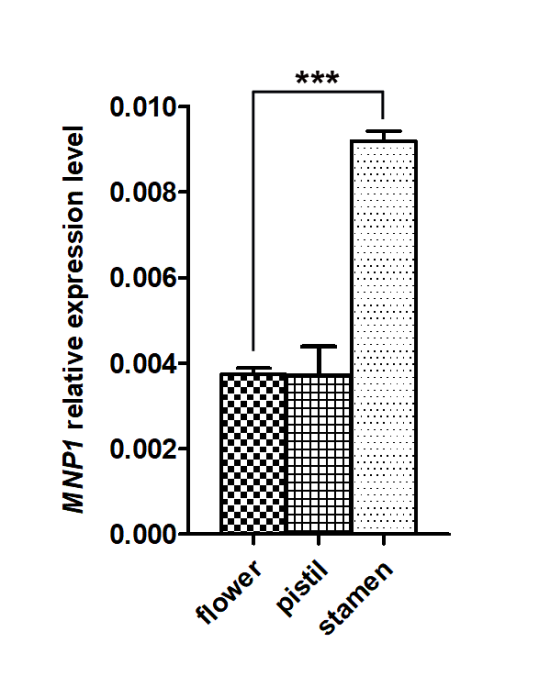


**Figure S7.** Relative expression of *MNP1/Medtr7g011663* in flower, pistil and stamen of WT. The samples are all mixed samples taken from various floral organ development stages. Two-sample *t*-test, ****P*<0.001.

**
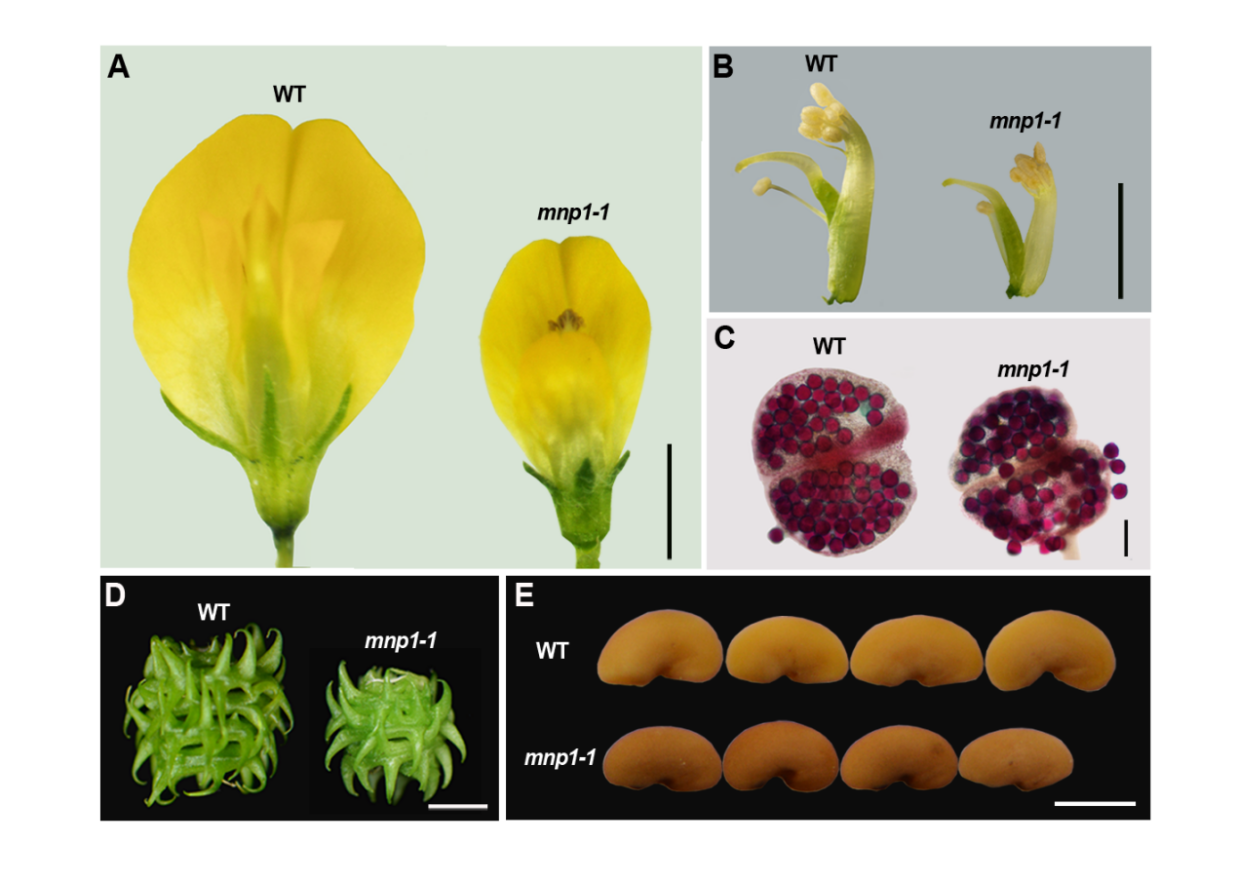
**

**Figure S8.** Fertility analysis of WT and *mnp1-1* mutant. **(A)** Flower. Scale bar =2 mm. **(B)** Stamens and pistil. Scale bar =2 mm. **(C)** Alexander’s staining of mature pollens. Scale bar =50 um. **(D)** Pod. Scale bar =3 mm. **(E)** Seeds. Scale bar =2 mm.

**Table S2.** The genes involved in this study.

| **Gene name** | **Protein name** | **Locus name** |
| --- | --- | --- |
| *MNP1*  *MtKS*  *MtKO*  *MtKAO1*  *MtKAO2*  *MtCYP714_A1*  *MtCYP714_A2*  *MtCYP714_C1*  *MtCYP714_C2*  *Mt20ox1*  *Mt20ox2*  *Mt20ox4*  *Mt20ox5*  *Mt20ox6*  *Mt20ox7*  *Mt20ox8*  *Mt3ox1*  *Mt3ox2*  *MtCYCB1;1*  *MtKNOLLE* | Copalyl diphosphate synthase  *Ent*-kaurene synthase  *Ent*-kaurene oxidase  *Ent*-kaurenoic acid oxidase  *Ent*-kaurenoic acid oxidase  Cytochrome P450  Cytochrome P450  Cytochrome P450  Cytochrome P450  Gibberellin 20-oxidase  Gibberellin 20-oxidase  Gibberellin 20-oxidase  Gibberellin 20-oxidase  Gibberellin 20-oxidase  Gibberellin 20-oxidase  Gibberellin 20-oxidase  Gibberellin 3-beta-dioxygenase  Gibberellin 3-beta-dioxygenase  Cyclin-dependent protein kinase CYCB1;1  Syntaxin-related protein KNOLLE | *Medtr7g011663*  *Medtr2g064295*  *Medtr2g105360*  *Medtr2g031930*  *Medtr2g031920*  *Medtr3g093530*  *Medtr2g061200*  *Medtr7g055793*  *Medtr8g008530*  *Medtr1g102070*  *Medtr3g096500*  *Medtr1g081840*  *Medtr3g088745*  *Medtr8g093980*  *Medtr6g464620*  *Medtr8g033380*  *Medtr2g102570*  *Medtr1g011580*  *Medtr5g088980*  *Medtr5g012010* |

**Table S3.** Primers used in this study.

| **(Prisic et al. 2007)Primer name** | **Sequence (5’ to 3’)** | **Function** |
| --- | --- | --- |
| *MNP1*-GT-F1/R1 | TACTGCTTGGGTTGCTCTTG/TGGCATGTGCTCTTCATTCTCA | *mnp1-1* genotyping |
| *MNP1*-GT-F2/R2 | CTGATGATGTCATGCCTTGTG/TTTGCATACCAAGGCACGTC | *mnp1-2* genotyping |
| *MNP1*-GT-F3/R3 | TGGAAGGGATGTTAGGCTTGG/CGTTCGAGCCGATCAACCAA | *mnp1-3* genotyping |
| *MNP1*-GT-F4 | GTGCTGTGTCCTTAGTTGCT | *Tnt1* genotyping in *mnp1-1* |
| TntR2 | AGTTGGCTACCAATCCAACAAGGA |  |
| TntF2 | TCTTGTTAATTACCGTATCTCGGTGCTACA | *Tnt1* genotyping in *mnp1-2* |
| *MNP1*-GT-R4 | ACAAGCTTCAACCATGCCTAAC |  |
| TntF2 | TCTTGTTAATTACCGTATCTCGGTGCTACA | *Tnt1* genotyping in *mnp1-3* |
| *MNP1*-GT-F3 | TGGAAGGGATGTTAGGCTTGG |  |
| *MtACTIN*-F/R | TCAATGTGCCTGCCATGTATGT/ACTCACACCGTCACCAGAATCC | qRT-PCR |
| *MNP1*-F | TCAAGATGGTTCATGGGGTG | qRT-PCR |
| *MNP1*-GT-R1 | TGGCATGTGCTCTTCATTCTCA |  |
| *MtKS*-F/R | TCCTTCTTCAACGTCGCCTC/GGATGGTGATCGGGAAGACC | qRT-PCR |
| *MtKO*-F/R | AGCATAGTCCAGTTCCGATTGTC/TCCGGCAGGAATATGATATCCT | qRT-PCR |
| *MtKAO1*-F/R | TGAATGCTGGTCATGAGTCTTCA/TCTTGTTCTTCCTTGGCCTTTT | qRT-PCR |
| *MtKAO2*-F/R | TGCCCTGGAAATGATCTTGCT/TGTCCATTGGCCTTGTGTGT | qRT-PCR |
| *MtCYP714_A1*-F/R | ACCCTGAATGGCAAGATCGT/CTTGACACAAAGGCTGCTGG | qRT-PCR |
| *MtCYP714_A2*-F/R | TCCACCGGAATCAAGCAACA/GCTCTGAGGATGCCATCTCC | qRT-PCR |
| *MtCYP714_C1*-F/R | GTTCCCGTGTCTGTCCTGG/AAAGGCCGGTGAATGACAGT | qRT-PCR |
| *MtCYP714_C2*-F/R | TTTGGAATGGGACCTCGTGT/CAACTCCATGGCCAGGTTCT | qRT-PCR |
| *MtGA20ox1*-F/R | ACACAGTTCATTTGGCCTGATG/CAACAACCTTTGATGCTTCCATT | qRT-PCR |
| *MtGA20ox2*-F/R | CACAGCCCAAGAGGAGCTAAAA/AGCTCAGCAGCACTTGCAATAG | qRT-PCR |
| *MtGA20ox4*-F/R | GCATTAGGGACAGGACCACATT/AACACATCAAGCCCTCCAACTT | qRT-PCR |
| *MtGA20ox5*-F/R | GGTACAGGCCCTCATTGTGATC/GAACCGTATGCCACTTGTTGTC | qRT-PCR |
| *MtGA20ox6*-F/R | CCGGGTTGGTGTATCAAAAGT/ACCCAAACTAATGGCCAATAGC | qRT-PCR |
| *MtGA20ox7*-F/R | GGTGACACTTTCATGGCTCTTTC/TGGACAAACCACCTTATCACCTT | qRT-PCR |
| *MtGA20ox8*-F/R | GATTTCAGGCAATTTGGGAGTGT/ACTCATCCCAAGAAGTTCCATGA | qRT-PCR |
| *MtGA3ox1*-F/R | GCAGCCATGCAACTCAACTC/GAACTTGCAGCCCGCTTATG | qRT-PCR |
| **Primer name** | **Sequence (5’ to 3’)** | **Function** |
| *MtGA3ox2*-F/R | CCATGTTGGAGCAATGCAACT/AGGGATGTGTCTGTGTGTGG | qRT-PCR |
| *TPMNP1*-GFP-F | acgggggactcttgaccatggttATGTCTACTCACTTCTCCACCCACT | *TPMNP1* cloning for *p35S::TPMNP1-GFP* |
| *TPMNP1*-GFP-R | AcgtcccgggttaaccctaggCCCTTTTCCTTCTCATCATCAATGACTAT |  |
| *TPMNP1*-GFP-F | acgggggactcttgaccatggttATGTCTACTCACTTCTCCACCCACT | *MNP1* cloning for *p35S::MNP1-GFP* |
| *MNP1*-GFP-R | gacgtcccgggttaaccctaggTAAACAACTTTATCAAAGAGAACTTTGG |  |
| *CPS1*-GT-F/R | CAGACCCGAGACAGTAACTGC/TCTCTACTCGAGGCAAGCTTG | *cps1* genotyping |
| LBb1.3 | ATTTTGCCGATTTCGGAAC | *T-DNA* genotyping in *cps1* |
| *CPS1*-GT-R | TCTCTACTCGAGGCAAGCTTG |  |
| *MNP1*-RT-F | CTCCACCCACTTCCACCTT | PCR/RT-PCR for detecting *MNP1* cds fragment |
| *MNP1*-RT-R | TGTTCACATTCGCCATCCT |  |
| *AtACTIN-*F/R | CAGTGGTCGTACAACCGGTATTG/TGCTGTGATTTCTTTGCTCATACG | RT-PCR |
